# Liquid Biopsies in Lung Cancer: Four Emerging Technologies and Potential Clinical Applications

**DOI:** 10.3390/cancers11030331

**Published:** 2019-03-07

**Authors:** Dimple Chudasama, Periklis Katopodis, Nick Stone, Jennifer Haskell, Hannah Sheridan, Benjamin Gardner, Howard Urnovitz, Ekkehard Schuetz, Julia Beck, Marcia Hall, James Barr, Cristina Sisu, Alexandra Rice, Andreas Polychronis, Vladimir Anikin, Emmanouil Karteris

**Affiliations:** 1 Department of Life Sciences, Brunel University, London UB83PH, UK; dimple.chudasama@brunel.ac.uk (D.C.); periklis.katopodis@brunel.ac.uk (P.K.); marcia.hall@nhs.net (M.H.); cristina.sisu@brunel.ac.uk (C.S.); 2 Division of Thoracic Surgery, The Royal Brompton & Harefield NHS Foundation Trust, Harefield Hospital, London UB9 6JH, UK; jgbarr85@gmail.com (J.B.); a.rice@rbht.nhs.uk (A.R.); 3 College of Engineering, Mathematics and Physical Sciences University of Exeter, Exeter EX4 4QF, UK; n.stone@exeter.ac.uk (N.S.); jmh251@exeter.ac.uk (J.H.); hs438@exeter.ac.uk (H.S.); B.Gardner@exeter.ac.uk (B.G.); 4 Chronix Biomedical, 37073 Göttingen, Germany; hbu@chronixbiomedical.de (H.U.); esc@chronixbiomedical.de (E.S.); jule_beck@mac.com (J.B.); 5 Mount Vernon Cancer Centre, Middlesex HA6 2RN, UK; andreas.polychronis@nhs.net; 6 Department of Oncology and Reconstructive Surgery, Sechenov First Moscow State Medical University, Moscow 119146, Russia

**Keywords:** liquid biopsies, circulating tumour cells, lung cancer, copy number instability, Raman spectroscopy

## Abstract

*Background:* Liquid biopsies offer a promising alternative to tissue samples, providing non-invasive diagnostic approaches or serial monitoring of disease evolution. However, certain challenges remain, and the full potential of liquid biopsies has yet to be reached. Here we report several methodological approaches to interrogate liquid biopsies using circulating tumour cell (CTC) enumeration and characterisation, transcriptomics, Raman spectroscopy, and copy number instability (CNI) scores using blood samples of lung cancer (LC) patients. *Methods*: We choose LC; since it still is the most common cause of cancer-related mortality worldwide, and therefore there is a need for development of new non-invasive diagnostic/prognostic technologies. Changes in gene expression were assessed using RNA-seq, and in CTCs using ImageStream, an imaging flow-cytometer. CNI scores, from paired tissue/ctDNA were also explored. Raman spectroscopy was used to provide chemical fingerprints of plasma samples. *Results*: CTCs were detected in all LC patients (*n* = 10). We observed a significant increase in CTC levels in LC patients (*n* = 10) compared to controls (*n* = 21). A similar CNI was noted in the tissue and plasma of 2 patients, where higher CNI scores corresponded with poorer outcome. Significant changes in Raman spectra (carotenoid concentrations) were noted in LC patients (*n* = 20) compared to controls (*n* = 10). RNA-seq revealed differential expression of 21 genes between LC cases and controls in both LC tissue and blood samples. *Conclusions*: Liquid biopsies can potentially provide a more comprehensive picture of the disease compared to a single tissue biopsy. CTC enumeration is feasible and sensitive for LC patients. Molecular profiling of CTCs is also possible from total blood. CNI scores and Raman spectra require further investigation. Further work is being undertaken to explore these methods of detection in a larger LC cohort.

## 1. Introduction

Over the past decade, the concept of liquid biopsies has been introduced as an alternative to a conventional needle (tissue) biopsies for several cancers. Several possible advantages have been recently discussed, including the use of different biofluids (blood, urine, sputum, saliva), early warning of pending resistance, serial monitoring of patients undergoing numerous cycles of treatment, less risk to patients, especially those who are not candidates for invasive biopsy [1,2]. For example, tests can be conducted on a blood sample (i.e., non-invasively) to examine circulating tumour cells (CTCs) or circulating tumour DNA (ctDNA) with the aim to provide a better insight into the real-time dynamics of a disease.

CTCs are tumour cells present in the bloodstream that shed either from the primary tumour or its metastasis [3,4]. These cells are different to normal circulating blood cells and express tumour-specific characteristics [5,6]. A sub-population of CTCs have the functional capacity to enter distant sites and progress towards metastatic events; termed disseminated tumour cells (DTCs) [7]. CTCs are of prognostic value in various cancers, but their clinical value is still under intense investigation [8,9,10].

Another area where advancements have been made is that of the use of ctDNA, which is fragmented DNA that originates directly from the cancer cell itself [11]. Emerging studies relate ctDNA to tumour progression and rate of tumour cell turnover, a biological measure of tumour aggressiveness [12,13]. In a recent study, we have demonstrated a potential use of Alu repeats ratios for prognostic purposes in the advanced setting for lung cancer (LC) patients [14].

We therefore hypothesised that the assessment of circulating blood markers as surrogate real-time biopsies for is possible. In this study, we employed a wide-repertoire of techniques to explore this possibility for patients suffering with LC. LC is still one of the biggest killers in the western world today, with incidence remaining high, and as many as 46,403 new cases reported in the UK [15,16]. Some 30–50% of non-small cell LC patients (NSCLC) treated with curative intent suffer recurrence, and variable response rates to chemotherapy drugs are hindering survival rates [17,18,19]. Thus, a routine biomarker monitoring system post-radical therapy could be of significant prognostic value, detecting earlier relapse and allowing rapid initiation of treatment. Such a biomarker system would also benefit patients undergoing therapies (i.e., radiotherapy, chemotherapy) as an alternate or additional method of monitoring and predicting response, rather than rely entirely on imaging. 

In this study, using a novel flow-cytometry imaging technology, we have successfully detected and characterised CTCs from LC patients, and identified an 18-gene signature based on RNA-seq analyses. We have also demonstrated that measuring the chemical composition plasma using Raman spectroscopy can be of diagnostic value. Finally, we provide evidence (based on CNI scoring of material from two patients) that their liquid biopsy is a true representative of all the genetic changes of their tumours.

## 2. Results

### 2.1. Identification of A549 and H1975 Lung Cancer Cells Mixed with Blood in Reconstruction Experiments Using AE1/AE3 

**Figure 1:**
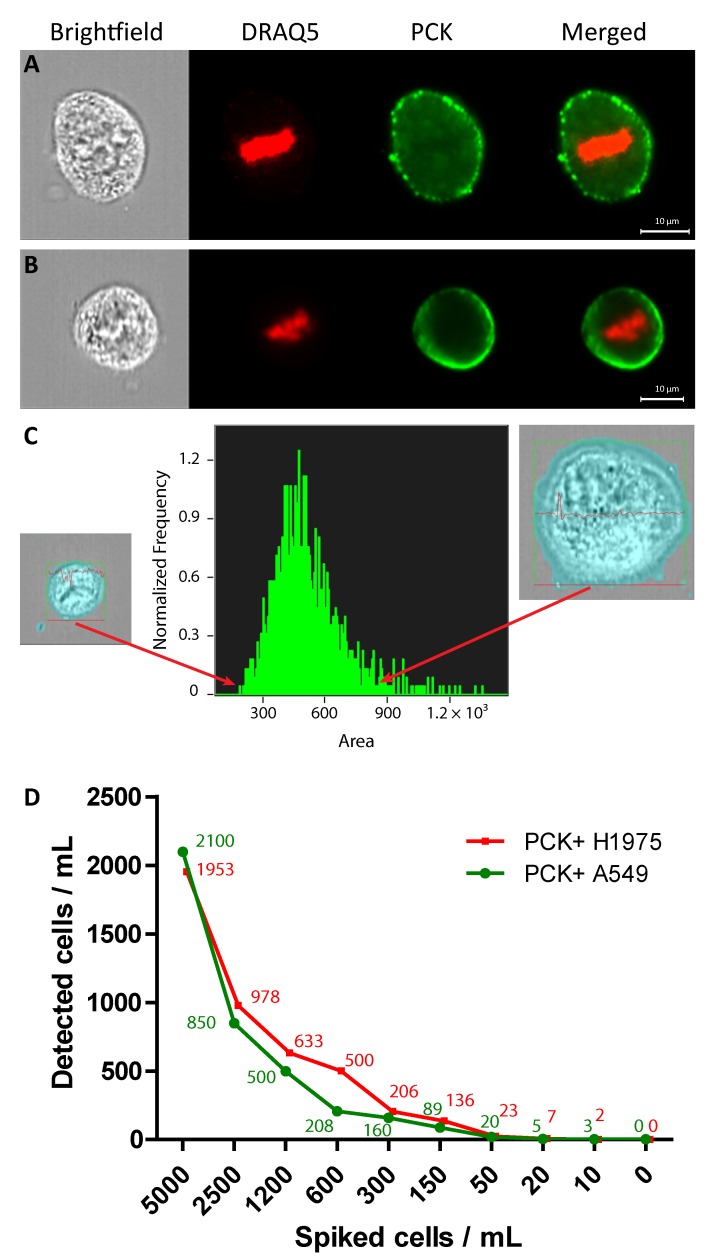
Positive AE1/AE3 staining is signified by a deep green halo encircling the entire A549 and H1975 cells’ cytoplasm. Positive A549 (Panel **A**) and H1975 (Panel **B**) cells are depicted as having a positive AE1/AE3 (green) staining in addition to positive DRAQ5 (red) staining of the nucleus. (Panel **C**) A small and a large H1975 AE1/AE3+ cells. The H1975 cell size is measured in pixels area and expands from 48 pixels = 12 μm diameter to 104 pixels = 26 μm diameter. Cell on the left are the smallest cells detected, whereas the cells on the right of the histogram are the largest H1975 cells observed in these samples. Both bright field images are highlighted with a blue mask as the software measured variable statistics for both cells and calculated their size. (Panel **D**) Serial dilutions of cell lines (A549 and H1975) spiked in 1 mL of healthy donor blood, retrieved based on AE1/AE3 (PCK+) staining.

We assessed the feasibility and efficiency of staining lung cancers cells with a pan-cytokeratin marker through a series of proof-of-principle experiments using the A549 and H1975 lung cancer cell lines. A549 is an epithelial hypotriploid LC human cell line widely used for in vitro studies in LC, with the modal chromosome number of 66 occurring in 24% of cells, and a known KRAS mutation. H1975, is an adenocarcinoma cell line with key mutations in *CDKN2A*, *EGFR, PIK3CA* and *TP53*. Cell pellets from these cell lines were stained with AE1/AE3 antibody; a pan cytokeratin marker used widely in NHS histopathology laboratories as part of routine cancer diagnostics [20]. For negative selection, the CD45 marker was used to exclude cells of hematopoietic origin. For the cytokeratin marker, a positive stain would resemble a green halo-like staining around the cell’s surface, whereas the leukocyte marker was orange due to the different conjugated fluophores; thus, enabling easy identification (Figure 1A,B). 

Previous studies have shown that ImageStream™ (AMNIS, Seattle, WA, USA)—a relatively new imaging flow-cytometry platform—allows objective characterisation of CTCs based on size [21]. We expanded our model, using whole blood spiked with the LC cell lines to mimic a liquid biopsy. We used this test to evaluate both staining in whole blood vehicle, and to prove ability to differentiate cancer cells from white blood cells (Figure 1C,D). We have also assessed the surface area of the AE1/AE3 positive A549 and H1975 cells: the cell size (area) was measured in pixels and ranged from 430 μm^2^ to 2650 μm^2^ for the A549 and 240 μm^2^ to 1368 μm^2^ for the H1975 (1 pixel = 0.25 μm^2^). 

Serial cell dilutions (150–5000) of both A549 and H1975 cell lines were added in 1 mL of whole blood and cell retrieval was quantified. The retrieval for 5000 A549 cells was 42%; for 2500 cells was 34%; for 1200 cells 41.6%; for 600 cells 50%; for 300 cells 63.8%; for 150 cells 65.2%, for 50 cells 40%, for 20 cells 25%, and for 10 cells 30%. No false AE1/AE3 positive were detected when no cells were added in 1 mL of healthy donor’s blood (Figure 1D).

For the H1975 cell line, the retrieval for 5000 was 39%; for 2500 cells was 39.1%; for 1200 cells 52.7%; for 600 cells 83.3%; for 300 cells 68.8%; for 150 cells 90.9%, for 50 cells 46%, for 20 cells 35%, and for 10 cells 20%. Similarly, no false AE1/AE3 positive were detected when no cells were added in 1 mL of healthy donor’s blood (Figure 1D).

### 2.2. CTC Enumeration and Characterisation of LC Patients Using ImageStream™

**Figure 2:**
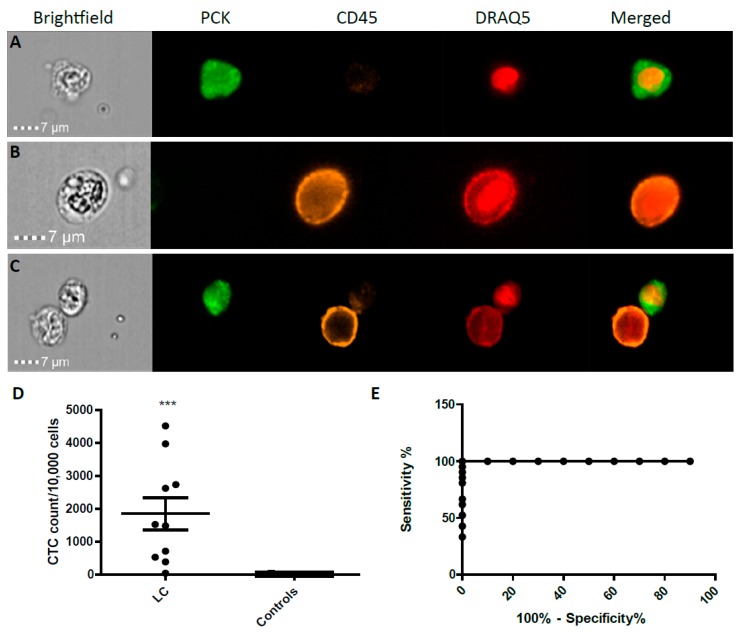
(**A**) A positive lung cancer AE1/AE3 CTC is depicted by a strong green staining around the cell, and a positive nuclear stain see in red; (**B**) A positive CD45 white blood cell (WBC) and negative for AE1/AE3. (**C**) AE1/AE3+/CD45− and AE1/AE3−/CD45+ cell in the same frame as visualised by the ImageStream™, magnification 60×. ImageStream™ CTCs quantification in blood of lung cancer patients vs. benign controls with AE1/AE3 antibody staining. CTC quantification was done per 10,000 cells. The lung cancer group showed substantially increased positive CTCs in comparison to the normal group, *** *p* < 0.001 (**D**). Diagnostic tool evaluation using ROC curve analysis of the CTC data from lung cancer samples was done to assess sensitivity and specificity. Strong positive sensitivity and specificity was shown in LC vs. normal, AUC 1.000 (*p* < 0.0001; Panel **E**).

All LC and control blood samples were analysed through the AMNIS^®^ ImageStream™ system. Designated LC CTCs had positive staining with AE1/AE3 (pan-cytokeratin) antibody and negative staining for CD45 with positive nuclear staining of DRAQ5 antibody. CTCs were quantified from blood samples of LC patients (early stage I–II, *n* = 8; and stage IV *n* = 2), prior to undergoing surgical resection (Figure 2A–C). We observed a significant enrichment in the cancer cohort CTC counts (*n* = 10) compared to benign controls (*n* = 21) (Figure 2D, *p* < 0.0007). Blood samples from cancer patients were compared to samples from benign controls to ensure discrimination of the test. Strong positive sensitivity and specificity was shown in LC patients vs. benign control patients; area under curve (AUC) 1.000 (*p*-value < 0.0001; Figure 2E). 

### 2.3. RNA-Seq from Whole Blood and Matched Tissues of LC Patients and Controls

Next, we supplemented the liquid biopsy interrogation framework with insights from transcriptome analysis. For this, we examined the RNA-seq data from three LC patients’ matched tissue and blood samples, with matched tissue and blood samples from two benign control patients. Sample quality was tested by comparing gene expression levels between any two patients. We observed a higher level of homogeneity in gene expression levels from blood compared to tissue (Supplementary Figures S1 and S2). Comparisons took place of differential expression patterns in LC patient tissue and blood samples vs. benign controls, as well tissue vs. blood for each of the three LC patients (Supplementary Figure S3). Three broad gene groups were identified: (i) genes significantly up- or down- regulated between the two investigated stages (*p* < 5 × 10^−5^) with an absolute log2 (fold change) higher than 2; (ii) genes showing a differential expression pattern (*p* < 0.05) and an absolute log2 (fold change) higher than 2; (iii) genes that do not show any statistical significant change in the expression level between the two stages.

**Figure 3:**
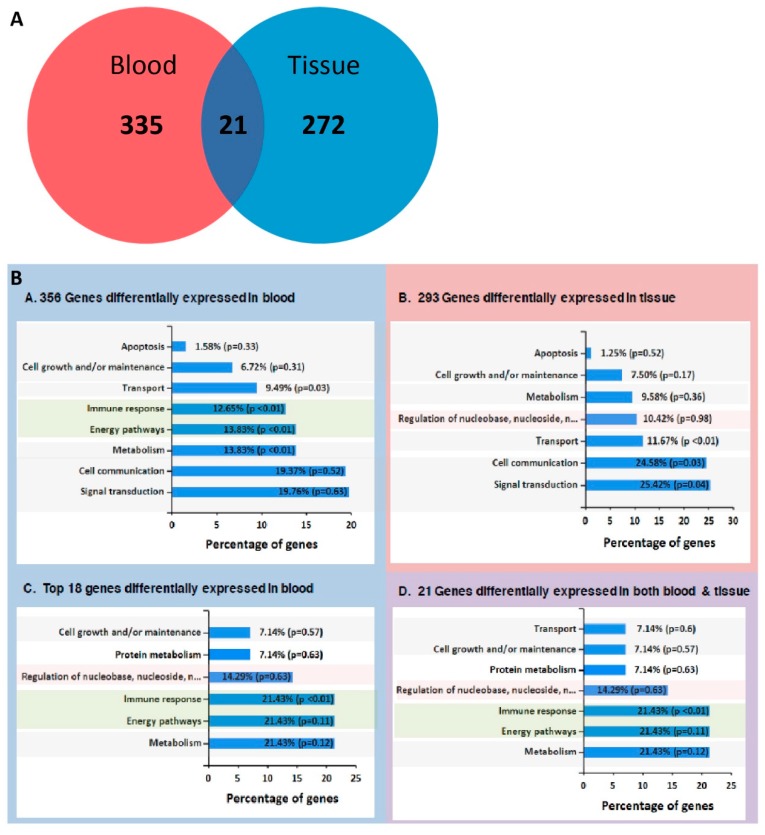
Venn diagram of differential expressed genes in blood and tissue. Panel (**A**): A total of 272 genes were shown to be differentially expressed in tumour tissue compared to controls, and 335 in cancer blood samples compared to controls. Twenty one genes show statistically significant differential expression pattern in both tissue and blood samples compared to control. Panel (**B**): Details on top GO terms resulting from functional enrichment analysis using Fun Rich software, for differentially expressed genes in blood (356), tumour tissue (293), blood only (18), and in both blood and tumour (21) compared to controls (Panels **A**–**D** in **B**).

Further analysis revealed 272 genes which are differentially expressed in LC tissue compared to benign control tissue, and 335 in LC blood samples compared to benign control blood samples (Figure 3A). Twenty one genes displayed significant differential expression between LC cases and benign controls in both tissue and blood samples, namely: *TTTY15, DDX3Y, DMKN, EIF1AY, KDM5D, C10orf9, TXLNGY, ELN, KRT77, RPS4Y1, SLC6A8, SPD1C, USP9Y, FRCLA, IGHV4-31, PRKY, ME132, XIST, ZFY, SORB5*, and *GSTT1*. We observed that while the majority of genes are down-regulated in patient samples compared to controls, there was only one gene that was over-expressed in both patient tissue and blood, with no or very little expression in both blood and tissue of control patients, X Inactive Specific Transcript (*XIST*). 

To expand our biomarker search horizon, we looked at genes that were overexpressed in patient blood compared to control. We filtered the differentially expressed genes in blood (335 + 21) based on following criteria: (i) have an expression level higher than 5 FPKM (minimal expression threshold for calling a protein coding gene transcribed [22]), (ii) show a statistical significant differential expression pattern with (iii) a log2 (fold change) > 2 in tumour vs. control. Using this selection workflow, we were able to identify 18 genes: *BTNL3, DEFA3, HLA-DQA2, KIAA1324, RPH3A, TMTC1, CD177, ECHDC3, HLA-DRB6, MSRB2, SERPING1, VNN1, CEBPE, GSTT1, IGHG2, NBPF14, THBS1, XIST*.

We functionally characterised the differentially expressed genes for the 335 (+21) and 272 (+21) genes in blood and tissue, respectively as well as the 18 genes known to be up-regulated in blood only using FunRich software (Figure 3B). The analysis showed an enrichment in immune response, energy pathway and metabolism functions for the 18 genes. It is interesting to point out that the top Gene Ontology (GO) terms enriched in the 18 genes match the top enriched terms in the set of 21 genes differentially expressed in both tissue and blood, highlighting that functional characteristics in genes from blood CTCs match those observed in tissue.

**Figure 4:**
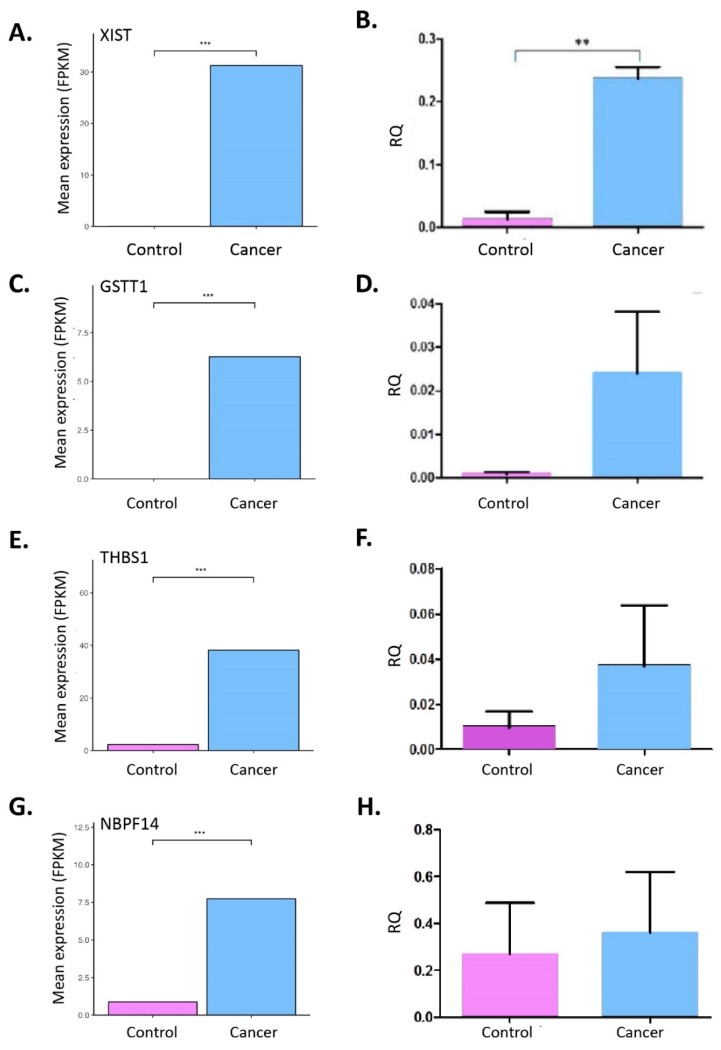
(**A**,**C**,**E**,**G**) show gene expression in blood samples from LC patients vs. control. Mean expression level is show in FPKM units, and was calculated using CuffDiff. (*** *p* < 0.001 computed using CuffDiff). (**B**,**D**,**F**,**H**) show qRT-PCR expression for XIST, GSTT1, THBS1 and NBPF14 respectively. Validation data shows the similar up-regulation of genes’ expression in patient blood samples compared to controls. ** *p <* 0.01.

We validated the RNA-seq expression analysis results using qPCR. Specifically, experiments were carried out on the four statistically significant over-expressed genes in blood from the 18 identified. These include XIST, GSTT1, THBS1 and NBPF14. These were tested in five patient blood samples. The qPCR results partially corroborated the data obtained from RNA-seq; since a clear trend of increased gene expression was noted for GSTT1, THBS1 but not for NBPF14 (Figure 4).

### 2.4. Copy Number Instability

We furthered our analysis by examining the changes at chromosomal level. The copy number instability (CNI) score is a general measure of genomic instability and is directly related to the regional chromosomal DNA ploidy. In this work we analysed paired tissue and DNA from plasma samples.

**Figure 5:**
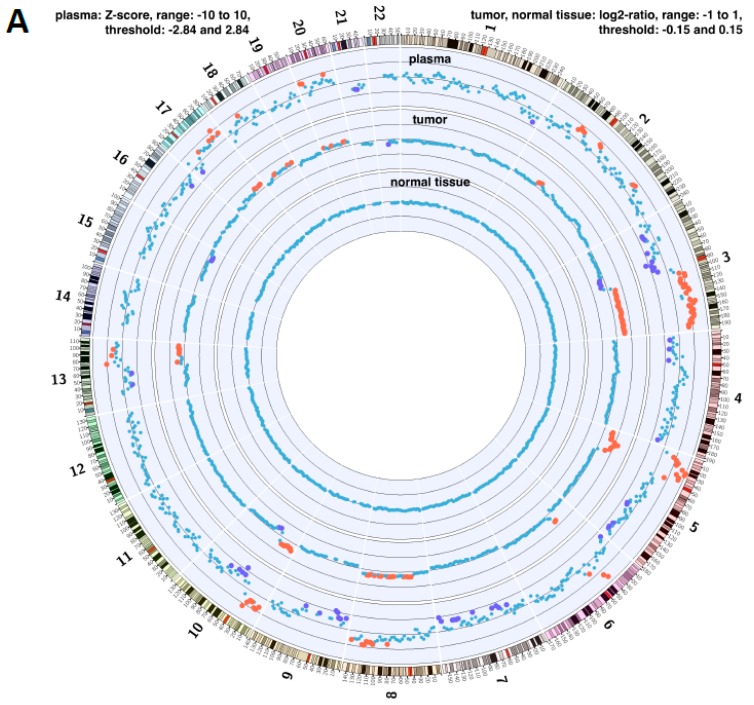

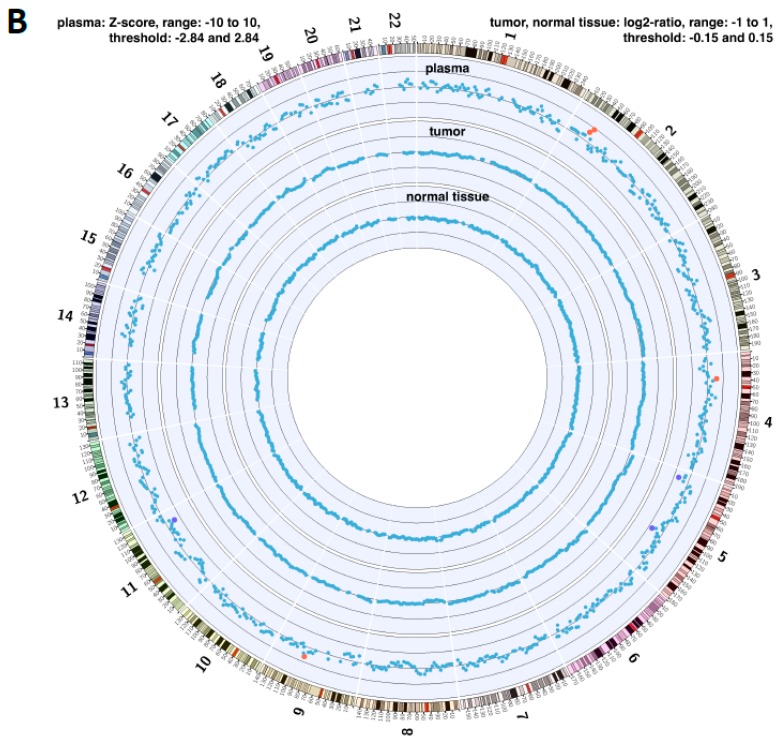
Circos plots for two lung cancer patients, highlighting the chromosomal CNI and corresponding chromosomal location. Patient in (Panel **A**) has extensive copy number changes, visualised by the red and purple dots (loss and gains based on values that are significantly different to normal). Patient in (Panel **B**) shows very little chromosomal copy number changes, and these can only be seen in the plasma.

Circos plots were generated using data from two lung cancer patients, highlighting the chromosomal copy number instability and corresponding chromosome and location. The first patient diagnosed with squamous cell carcinoma shows extensive copy number changes, visualised by the red and purple dots (loss and gains based on values that are significantly different to normal); the patient died 60 days post-operatively (Figure 5A). The copy number changes are identical and occur in the same regions in both tumour tissue and plasma samples. This result showcases liquid biopsy as an excellent mirror of changes observed in tissue. By contrast, the second patient diagnosed with adenocarcinoma LC, has very few chromosomal copy number changes and only in the plasma (ctDNA). This LC patient is still alive 397 days post-operatively (Figure 5B). CNI from LC tissue may be have prognostic value for pre-operative patients with early stage LC.

### 2.5. Raman Spectroscopy 

**Figure 6:**
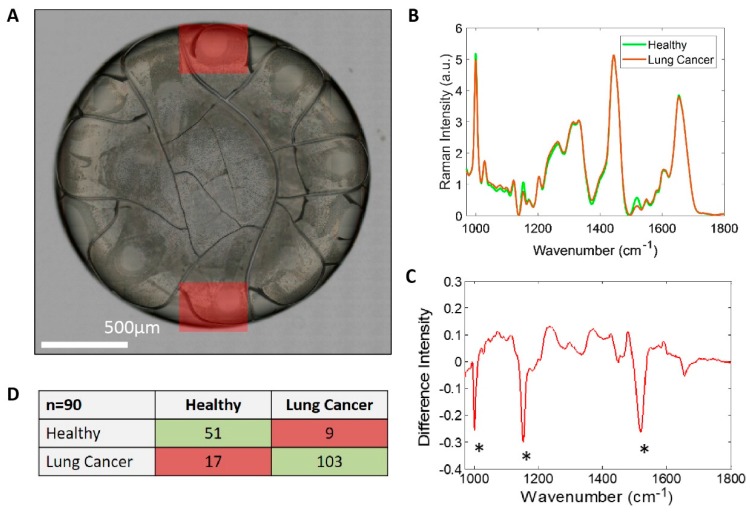
(**A**): 50× white light image of a 1 μL drop of blood plasma deposited on polished stainless steel, red regions indicating approximate position and area of Raman maps. (**B**): Combined map average Raman spectra of healthy control blood plasma drops (green) and lung cancer (red). (**C**): Control subtracted lung Raman spectrum. (**D**) Confusion matrix demonstrating the performance of the cross-validated PCA-LDA model for pathology classification. In benign controls (Healthy) 51 out of 60 measurements were predicted correctly (green); whereas only nine were false positives (red). In LC patients, 103 out of 130 measurements were predicted correctly (green) whereas only 17 measurements were mispredicted (red).

The Raman study of dried plasma (Figure 6A) set out to measure the biochemical fingerprint from the pre-concentrated coffee ring to assess if Raman could be used as a tool for discrimination of patients with and without lunch cancer. This technique has therefore the potential to detect molecular changes prior to any morphological ones occurring in the tissue, offering thus many possibilities to aid the early detection of cancers. The non-invasive nature as well as high specificity of Raman spectroscopy suggests its potential use in differentiating benign and malignant tissues, offering an alternative to conventional biopsies [23]. 

As can be seen (Figure 6B) small differences are observed in the Raman spectra between the control group (*n* = 10) and the lung cancer cohort (*n* = 20), which is made more apparent in the control subtracted spectrum (Figure 6C). Figure 6C, shows a pronounced decrease in three key Raman bands as highlighted, which can be tentatively assigned to belonging to carotenoids. The results of the PCA-LDA classification model (Figure 6D) demonstrate that it was possible to achieve high sensitivity (86%) and specificity (85%), for correct classification of LC or benign control using Raman spectra acquired from drops of plasma.

## 3. Discussion

In this study, we demonstrate using a wide repertoire of techniques that liquid biopsy can become a valuable alternative tool when it comes to cancer screening. We provide evidence that characterisation, and quantification of CTCs in the blood of LC patients without enrichment is possible, using the ImageStream™ technology. Our findings corroborate previously published reports in terms of presence of CTCs in LC [24,25,26]. However, we were able to identify substantially more CTCs per patient without enrichment (apart of removal of RBCs) compared with published techniques where EpCAM is utilised to enrich blood samples for CTCs [27,28]. One caveat of these approaches is that EpCAM-based enrichment might miss CTCs undergoing epithelial to mesenchymal transition (EMT) [29]. Current enrichment methods vary markedly in their approaches of CTC isolation and characterisation: from size- and gradient-based to dielectrophoresis and use of surface-based antigens like the EpCAM CellSearch™ System (Menarini-Silicon Biosystems, Castel Maggiore (BO), Italy), is the only FDA-approved method that monitors CTC levels in patients with prostate, breast and colorectal cancers. This platform uses ferrofluid nanoparticles containing EpCAM antibodies to capture CTCs, that are then further verified by cytokeratin staining [30]. 

Moreover, there is still controversy around the actual numbers of CTCs in cancer patients. For example, in a meritorious review article on the prognostic utility of CTCs, the authors mention an enormous range of CTCs in LC patients (range 0–5986 cells per ml of blood) [31]. Furthermore, a study that used a microcavity array (MCA) system that is label-free, the authors detected up to 2329 CTCs in a blood sample of LC patients [32]. We also acknowledge that there are differences in CTC enumeration to previous published studies [33]. This could stem out of experimental procedures (i.e., choice of collection tubes, handling of blood samples, their half-life in blood, choice of antibodies, use of IDEAS software) as well as interpatient variation that is well documented in LC. To date no standardisation has been achieved yet due to a plethora of different isolation and characterisation platforms, so further studies are also needed to compare sensitivity and specificity. 

We would also like to put forward another possibility of large numbers detected. Recently, it has been shown that circulating endothelial cells (CECs) may be useful for the assessment of NSCLC patients [34]. Interestingly, high numbers of CECs appear to present in cancer patients raising the possibility of cytokeratin staining detecting more than one type of cells; apart from WBCs. We appreciate this might be a limitation of our study but provides preliminary evidence for a far wider repertoire of studies to dissect the nature of these cells. Therefore, we would like to propose to the scientific community working in liquid biopsies to use the term “circulating tumour-related cells” rather “circulating tumour cells” until a bona fide universal description is adopted. Collectively, our data demonstrate a potential use of CTCs as a non-invasive screening tool in support of early diagnosis in LC. 

Genomic analysis in the form of ctDNA or total RNA is another growing area of interest in the field of cancer diagnosis and/or prognosis. Exploration of RNA-seq data from the blood and tissue of LC patients and benign control patients revealed 21 genes matching strict criteria for potential biomarkers (all exhibited similar differential expression patterns in both blood and tissue in LC patients versus benign control patients). Interestingly, X inactive-specific transcript (XIST)-a non-coding RNA gene- shows a marked up-regulation in cancer patients for both blood and tissue compared to benign control patients [35]. 

Long non-coding RNAs are known to often contribute to unrestricted growth and invasion of cancer cells, with XIST shown to be up-regulated in several cancers, including colorectal, gastric, non-small cell lung cancer (NSCLC), [36,37,38]. Silencing of the XIST gene resulted in suppressive functions, including, inhibition of cell proliferation and invasion, and induced apoptosis [39]. These findings suggest that XIST can act as a potential biomarker and/or target for therapeutic interventions [37]. 

Another liquid biopsy technique explored here was that of Raman spectroscopy. This is a method of measuring the biochemical composition of a material using the inelastic scattering of laser light [40,41]. Drop coated deposition Raman spectroscopy (or DCDRS) has been demonstrated to provide protein quantification [42] from body fluids such as tears [43] and blood [44] and be effective in identifying colon cancer [45]. In the present analysis we observed a significant loss in carotenoids in LC patients compared to controls. Carotenoids are a structurally and functionally diverse group of natural pigments of the polyene type, known to be very efficient physical and chemical quenchers of both singlet oxygen (^1^O_2_), and potent scavengers of other reactive oxygen species (ROS) [46]. Furthermore, it has been proposed that carotenoids such as β-cryptoxanthin stimulate the expression of an anti-oncogene, and *p73* (a p53-related gene) [47]. Thus, this observed loss in carotenoids could be regarded as a blood-based biomarker of tumorigenesis in LC patients. 

These findings are similar to the metabolomic differences observed between primary breast cancer cell lines compared to normal cells, using Raman spectroscopy [48]. Differences in the biochemical and structural make-up of tumour tissue were also noted in melanoma patients, allowing identification of specific components affected in this cohort [23,49]. Furthermore, with the complimentary technique of Fourier transform infrared spectroscopy on sputum it has been shown that biochemical differences are apparent in LC patients [50]. 

**Figure 7:**
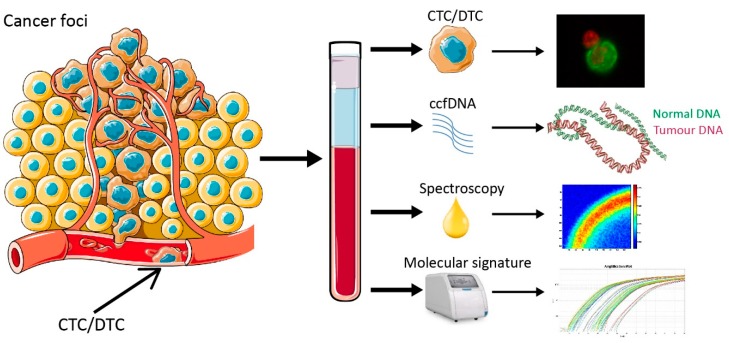
Different types of assessment from a single liquid biopsy. CTC: circulating tumour cells, DTC: disseminated tumour cells, ccfDNA: circulating cell-free DNA.

We acknowledge that our study has several limitations. One of them, frequently encountered in clinical studies is inter-patient variability [51]. Inter-patient variation might mask trends in gene and protein changes, particularly if these changes are subtle. This was evident in the CTC levels of LC patients. As highlighted earlier, the exact mechanism of CTC shedding is not well understood [52,53], and the frequency of CTCs as well as size seems to differ from patient to patient. A larger cohort of patients, including LCs from all different stages will provide a better insight. Moreover, use of more antibodies like CK7 or TTF-1 will also enable us to characterise CTCs from the entire pool of circulating cells detected. We also intend to correlate CNI, Raman spectra and CTC levels in a larger cohort of patients undergoing chemotherapy with a view to see if these readouts can be of any prognostic value. With the advancement of isolation technologies, it will also be interesting to obtain molecular and CNI profiling from isolated single CTCs. Collectively our data provide a broader repertoire of tests that can be performed from a single liquid biopsy (Figure 7). This approach can potentially facilitate the development of novel therapeutics and provide new insights in to the mechanisms and biology of the disease beyond LC [54,55,56].

## 4. Materials and Methods

### 4.1. Sample Collection and Preparation

Tissue and blood samples were collected from patients undergoing tissue biopsies/surgical resections for benign lung conditions and LC. Lung tissue was collected from Harefield Hospital, London, UK (Table 1, Table 2 and Table 3). All subjects gave their informed consent for inclusion before they participated in the study. The study was conducted in accordance with the Declaration of Helsinki, and the protocol was approved by the local Ethics Committee (IRAS number 151666). All tissue samples were retrieved in formalin fixed, paraffin embedded blocks. Tissue sections were cut at five µm using a microtome, followed by oil immersion to remove paraffin. 40 milligrams of lung tissue were lysed in a Tissue Lyser II (Qiagen, Manchester, UK) for two minutes with a three mm stainless steel ball bearing. Resulting RNA was stored at −80 °C. Blood samples were collected into EDTA tubes, inverted 10 times, and processed within four hours of acquiring. RNA was extracted from 0.5 mL of whole blood using the Ribopure purification kit, blood (ThermoFisher, Waltham, MA USA), according to the manufacturers’ instructions. Plasma was obtained from whole blood samples by centrifuging at 2500 rpm. The resulting plasma layer (~2 mL) was subjected to a further spin at 2500 rpm to remove any impurities. The extracted plasma sample was then stored at −80 °C and 1 mL of plasma was used for cfDNA extraction.

### 4.2. Cell Lines

A549 (ATCC^®^ CCL-185™) and H1975 (ATCC^®^ CRL-5908™) human adherent epithelial cells were used as in vitro models of human LC. A549 cells were grown in complete DMEM (Dulbecco’s Modified Eagle’s Medium, ThermoFisher, Waltham, MA, USA) with 10% fetal bovine serum (FBS, Gibco, ThermoFisher), 1% penicillin/streptomycin (Pen/strep) (Gibco) and 1% L-glutamine (Gibco). H1975 cells were grown in RPMI + 1% L-Glutamine (Gibco), 10% FBS and 1% Pen/strep. Cell lines were cultured at 37°C, 5% carbon dioxide (CO_2_) and subcultured when approached 80% confluency, approximately two times a week. Cell suspension was resuspended in different cell concentrations which were 5000, 2500, 1200, 600, 300 and 150 cells/mL and spiked in 1 mL blood from a donor.

### 4.3. ImageStream Processing and Analysis

One mL of whole blood per patient was treated with nine ml of red blood cell lysis buffer (RBC, G Biosciences, St. Louis, MO, USA), inverted eight times and incubated for 10 min with gentle agitation. The solution was then spun for 10 min at 2500 rpm and the supernatant removed. Then 3 mL of RBC lysis buffer was added to resuspend the pellet and another 10 min incubation and 10 min of spinning followed. The resulting pellet was then resuspended in 1 mL of ice cold 4% paraformaldehyde (PFA, Sigma-Aldrich, Gillingham, UK) and transferred in a 1.5 mL microcentrifuge tube for 5 min in ice. All the centrifuge steps following were for 3.5 min at 3500 rpm. The cell suspension was centrifuged and the PFA was removed. The pellet was washed with Phosphate-buffered saline (PBS) and centrifuged, and the PBS was removed. Samples were then blocked for one hour in 10% blocking buffer (10% Bovine Serum Albumin (BSA) in PBS; Sigma-Aldrich, Gillingham, UK). 1:100 conjugated AE1/AE3 antibody and 1:100 conjugated CD45/LCA PE-Texas Red^®^ (Life Technologies, Carlsbad, CA, USA), in BSA at 4 °C overnight in gentle agitation. Following the overnight incubation, the cells were spun and washed with washing buffer (0.1% Tween in PBS) to remove any remaining antibody. 

Washing buffer was removed and resuspended in 100 μL Accumax (Innovative Cell Technologies, San Diego, CA, USA) to dissociate any cellular aggregates. 0.5% of DRAQ5 DNA (Biostatus Ltd., Loughborough, UK) was added for nuclear stain before the visualisation on the ImageStream™. All the data files were then analysed on the IDEAS software^®^ Cells (AMNIS, Seattle, WA, USA) were gated using the intensity of the staining and their size. Samples positive for the AF488 (green), negative for CD45 (orange) and positive for the DRAQ5 (Biostatus Ltd.) nuclear stain (red), were classed as CTCs. Cells were quantified per 10,000 captured cells. 

### 4.4. Raman Spectroscopy

For each sample three replicate 1 μL drops of blood plasma were deposited onto a stainless-steel substrate and were allowed to fully dry under RTP conditions [57,58]. Two Raman maps (~300 × 300 μm) were collected from each drop in Streamline^®^ mode using an InVia^®^ system (Renishaw, Wotton-under-Edge, UK) under the following parameters: λ_ex_ 830 nm, 130 mW, 50× long working objective, 600 L/mm grating and a 3 s exposure time. In total, 120 Raman maps (20 patients by three drops by two maps = 120) were collected for LC, and 60 for the control samples (10 participants by three drops by two maps = 60). Each Raman map was averaged, baseline subtracted, and all data was normalised using standard normal variate (SNV) approach. This was to take into account any variations in signal that may have originated from subtle differences in focussing or the thickness of dried rings. Raman spectra contain myriad of information on biomolecules present. To ensure we did not discard information of use for discrimination, we included all the spectral data in the analysis, i.e., we did not preselect any known biochemical peaks in advance. To ensure independent testing of any classification models developed here, we left all the data from each participant (i.e., 6 mean spectra from the 6 maps) out of the model in turn. 

We then performed multivariate analysis in the form of principal component analysis (PCA) initially to reduce the dimensions of the data by calculating the most significant spectral variance described in the training dataset (all the data not including the left out participant’s data) and using a small number of scores to describe the relative contribution to each spectrum of each of the spectral components described in the PCA. We then used the PCA score values for each spectrum in a supervised classification approach, linear discriminant analysis (LDA) to calculate a single discrimination function combining the key contributions for discrimination from the PCA scores. This minimised the separation within samples from the same pathology groups and maximized the separation between them. With 30 participants there were 30 training models calculated from the remaining 29 participants and the pathology of the left out participant was prediction using the classification model. This is called leave-one-out cross-validation. The results show the prediction of pathology for each individual mean spectrum, i.e., six from each participant [59,60]. 

### 4.5. Chromosome Number Instability Scoring and Analysis

cfDNA was extracted from 2 mL of plasma using the Large Volume Viral Nucleic Acids Extraction Kit (Roche, Basel, Switzerland) according to the manufacturer’s instructions, but without addition of carrier RNA. Extracted cfDNA samples were processed using the ThruPLEX DNA-seq Kit (Takara Bio USA, Mountain View, CA, USA) according to the manufacturer’s instructions using dual-indexed adapters. The resulting sequencing libraries were pooled and paired-end sequenced (38bp/37bp) on a NextSeq500 (Illumina, Cambridge, UK). DNA from fresh tissue was extracted using the DNeasy Blood & Tissue Kit (Qiagen) and DNA from FFPE tissue was extracted using the GeneRead DNA FFPE Kit (Qiagen). Sequencing libraries from tissue DNA were constructed using the NEBNext Ultra II DNA Library Prep Kit for Illumina (New England Biolabs, Ipswich, MA, USA). CNI Scores were assessed as described [55,61]. Briefly, shotgun sequencing libraries were prepared from the extracted cfDNA and sequenced at low coverage (∼30 M reads per sample). The copy-number differences are detected by read counting statistics after mapping to the reference genome (HG19). Read counts were transformed into log2 transformed read ratios were obtained for 701 windows (each ∼5.5 Mbp) distributed over the whole genome and converted into Z values using values obtained from a reference group of 133 normal cfDNA samples. For the windows in which the null hypothesis (equality to reference) is rejected at a 0.2% false-positive rate, the absolute values of the Gaussian cumulative density function are summed to generate the CNI, thus reflecting the number and the amplitude of aberrations in the tumour as well as the tumour fraction of cfDNA. 8.6 ng for MAS4 and 4.1 ng for MAS12 was the cfDNA was input into the library preparation for the CNI experiments.

Circos plots show the data of the CNI analyses; each dot represents a genomic bin for which the copy-number was calculated. Plasma: Values that are significantly different from normal individuals are displayed as red (gain)/purple (loss) dots. Tumour: Read counts per bin were normalized to the median read counts over all bins. Ratios are displayed as log2 values, whereby log2 values > 0.15 (gain) or < –0.15 (loss) are displayed as red or purple dots.

### 4.6. RNA-Seq Processing and Analysis

Extracted RNA samples from matched blood and tissue of LC patients (*n* = 3) and controls (*n* = 3) were sent to the Wellcome Trust Genomic Centre (Oxford University, Oxford UK) for RNA-sequencing. RNA samples were normalised to 630 ng total RNA and the libraries prepared with the Illumina TruSeq Stranded mRNA Library Prep Kit (Illumina, Cambridge, UK) which involves isolation of the polyA containing mRNA molecules using poly-T oligo attached magnetic beads. All libraries were pooled equimolar and sequenced on one lane of HiSeq4000 at 75 bp paired end according to Illumina specifications.

The RNA-seq data was analysed using open source software from the Tuxedo suite: namely TopHat2 [62] and Cufflinks [63]. The paired end raw reads were mapped to the human reference genome hg37 (Ensembl 74) using the annotations from GENCODE 19 [22], withTopHat2 (Bowtie 2) under standard conditions. The resulting alignments were filtered for high quality hits using Samtools [64] with a minimum selection threshold score of 30. Next, we used Cufflinks to assemble the mapped reads into transcripts and quantify their expression levels in patient and control samples. Finally, we used Cuffdiff, as part of the Cufflinks package, to identify differentially transcribed genes and transcripts between any two states (cancer tissue vs. normal, cancer blood vs. normal, cancer tissue vs. cancer blood, and normal tissue vs. normal blood). Functional enrichment analyses and Venn diagrams were performed in the open software FunRich [65]. The statistical cut-off of functional enrichment analyses using this stand-alone software was kept at default setting with a *p*-value < 0.05 after Bonferroni correction [66].

### 4.7. Statistical Analyses

Statistical analyses were performed using one-way ANOVA followed by Tukey’s Multiple Comparison Test with significance determined at the level of *p* < 0.05.

## 5. Conclusions

In summary, liquid biopsies can potentially provide a more comprehensive picture of the disease compared to a single tissue biopsy. Enumeration and characterization of cancer-related circulating cells is feasible and sensitive for LC patients. Molecular profiling (RNAseq) is also possible from total blood. CNI scores and Raman spectroscopy require further investigation for their use as diagnostic/prognostic tests.

## Figures and Tables

**Table 1:** LC patient demographics for the CTC study.

Pre-Operative Characteristics	*n*	Percentage
Male/Female	6	46.2
Age at surgery (median)	70 years (range 34–79)	-
BMI (median)	21.1	-
Smoker (current or ex)	11	84.6
Diabetes	3	23.1
IHD	5	38.5
**Surgical resection**		
Wedge	2	15.4
Segmentectomy	1	7.7
Lobectomy	9	69.2
Bilobectomy	1	7.7
**Surgical approach**		
VATS	4	30.8
Thoracotomy	9	69.2
**Stage (lung cancer patients)**		
I	3	30.0
II	5	50.0
III	0	0.0
IV	2	20.0
**Histology**		
Lung squamous cell carcinoma	2	15.4
Lung adenocarcinoma	8	61.5
Metastatic adenocarcinoma	3	23.1
**Survival**		
Dead	6	46.2
Alive	7	53.8
Median survival, months	22.8 (range 0.6–24.8)	-

LC: Lung Cancer, CTC: Circulating Tumour Cells, BMI: Body Mass Index, IHD: Ischemic Heart Disease, VATS: Video-assisted thoracoscopic surgery.

**Table 2:** LC patient demographics for the Raman spectroscopy study.

Pre-Operative Characteristics	*n*	Percentage
Male/Female	12	60.0
Age at surgery (median)	70 years (range 34–79)	-
BMI (median)	21.6	-
Smoker (current or ex)	15	75.0
Diabetes	3	15.0
IHD	5	25.0
**Surgical resection**		
Wedge	2	10.0
Segmentectomy	1	5.0
Lobectomy	15	75.0
Bilobectomy	1	5.0
Pneumonectomy	1	5.0
**Surgical approach**		
VATS	6	30.0
Thoracotomy	14	70.0
**Stage (lung cancer patients)**		
I	3	15.0
II	9	45.0
III	2	10.0
IV	2 (10.0%)	
**Histology**		
Lung squamous cell carcinoma	8	40.0
Lung adenocarcinoma	8	40.0
Metastasis	4	20.0
**Survival**		
Dead	7	35.0
Alive	13	65.0
Median survival, months	26.2 (range 0.6–28.9)	-

**Table 3:** Benign control demographics.

Control Characteristics	*n*	Percentage
Male/Female	8	38.0
Age at surgery (median)	49 years (range 28–70)	-
**Control type**		
Healthy volunteer	18	86.0
Bullectomy	3	14.0

## Supplementary Materials

The following are available online at https://www.mdpi.com/2072-6694/11/3/331/s1, Figure S1: Cross patients’ gene expression consistency, Figure S2: Cross control samples gene expression consistency, Figure S3: RNAseq gene expression analysis in tissue and blood.

## References

[1] Khoo B.L., Grenci G., Jing T., Lim Y.B., Lee S.C., Thiery J.P., Han J., Lim C.T. (2016). Liquid biopsy and therapeutic response: Circulating tumor cell cultures for evaluation of anticancer treatment. Sci. Adv..

[2] Huang W.-L., Chen Y.-L., Yang S.-C., Ho C.-L., Wei F., Wong D.T., Su W.-C., Lin C.-C. (2017). Liquid biopsy genotyping in lung cancer: Ready for clinical utility?. Oncotarget.

[3] Cristofanilli M., Hayes D.F., Budd G.T., Ellis M.J., Stopeck A., Reuben J.M., Doyle G.V., Matera J., Allard W.J., Miller M.C. (2005). Circulating tumor cells: A novel prognostic factor for newly diagnosed metastatic breast cancer. J. Clin. Oncol..

[4] Hou H.W., Warkiani M.E., Khoo B.L., Li Z.R., Soo R.A., Tan D.S.-W., Lim W.-T., Han J., Bhagat A.A.S., Lim C.T. (2013). Isolation and retrieval of circulating tumor cells using centrifugal forces. Sci. Rep..

[5] Park J.-M., Lee J.-Y., Lee J.-G., Jeong H., Oh J.-M., Kim Y.J., Park D., Kim M.S., Lee H.J., Oh J.H. (2012). Highly Efficient Assay of Circulating Tumor Cells by Selective Sedimentation with a Density Gradient Medium and Microfiltration from Whole Blood. Anal. Chem..

[6] Krebs M.G., Hou J.-M., Sloane R., Lancashire L., Priest L., Nonaka D., Ward T.H., Backen A., Clack G., Hughes A. (2012). Analysis of circulating tumor cells in patients with non-small cell lung cancer using epithelial marker-dependent and -independent approaches. J. Thorac. Oncol..

[7] Dasgupta A., Lim A.R., Ghajar C.M. (2017). Circulating and disseminated tumor cells: Harbingers or initiators of metastasis?. Mol. Oncol..

[8] Kapeleris J., Kulasinghe A., Warkiani M.E., Vela I., Kenny L., O’Byrne K., Punyadeera C. (2018). The Prognostic Role of Circulating Tumor Cells (CTCs) in Lung Cancer. Front. Oncol..

[9] Chudasama D., Burnside N., Beeson J., Karteris E., Rice A., Anikin V. (2017). Perioperative detection of circulating tumour cells in patients with lung cancer. Oncol. Lett..

[10] Chudasama D., Freydina D.V., Freidin M.B., Leung M., Montero Fernandez A., Rice A., Nicholson A.G., Karteris E., Anikin V., Lim E. (2016). Inertia based microfluidic capture and characterisation of circulating tumour cells for the diagnosis of lung cancer. Ann. Transl. Med..

[11] Huang A., Zhang X., Zhou S.-L., Cao Y., Huang X.-W., Fan J., Yang X.-R., Zhou J. (2016). Plasma Circulating Cell-free DNA Integrity as a Promising Biomarker for Diagnosis and Surveillance in Patients with Hepatocellular Carcinoma. J. Cancer.

[12] Fournie G.J., Courtin J.P., Laval F., Chale J.J., Pourrat J.P., Pujazon M.C., Lauque D., Carles P. (1995). Plasma DNA as a marker of cancerous cell death. Investigations in patients suffering from lung cancer and in nude mice bearing human tumours. Cancer Lett..

[13] Diehl F., Schmidt K., Choti M.A., Romans K., Goodman S., Li M., Thornton K., Agrawal N., Sokoll L., Szabo S.A. (2008). Circulating mutant DNA to assess tumor dynamics. Nat. Med..

[14] Chudasama D.Y., Aladag Z., Felicien M.I., Hall M., Beeson J., Asadi N., Gidron Y., Karteris E., Anikin V.B. (2018). Prognostic value of the DNA integrity index in patients with malignant lung tumors. Oncotarget.

[15] (2018). Lung Cancer Incidence Statistics.

[16] Wong M.C.S., Lao X.Q., Ho K.-F., Goggins W.B., Tse S.L.A. (2017). Incidence and mortality of lung cancer: Global trends and association with socioeconomic status. Sci. Rep..

[17] Al-Kattan K., Sepsas E., Fountain S.W., Townsend E.R. (1997). Disease recurrence after resection for stage I lung cancer. Eur. J. Cardiothorac. Surg..

[18] Uramoto H., Tanaka F. (2014). Recurrence after surgery in patients with NSCLC. Transl. Lung Cancer Res..

[19] Carnio S., Novello S., Papotti M., Loiacono M., Scagliotti G.V. (2013). Prognostic and predictive biomarkers in early stage non-small cell lung cancer: Tumor based approaches including gene signatures. Transl. Lung Cancer Res..

[20] Travis W.D. (2012). Update on small cell carcinoma and its differentiation from squamous cell carcinoma and other non-small cell carcinomas. Mod. Pathol..

[21] Ogle L.F., Orr J.G., Willoughby C.E., Hutton C., McPherson S., Plummer R., Boddy A.V., Curtin N.J., Jamieson D., Reeves H.L. (2016). Imagestream detection and characterisation of circulating tumour—A liquid biopsy for hepatocellular carcinoma?. J. Hepatol..

[22] Harrow J., Frankish A., Gonzalez J.M., Tapanari E., Diekhans M., Kokocinski F., Aken B.L., Barrell D., Zadissa A., Searle S. (2012). GENCODE: The reference human genome annotation for The ENCODE Project. Genome Res..

[23] Mazurenka M., Behrendt L., Meinhardt-Wollweber M., Morgner U., Roth B. (2017). Development of a combined OCT-Raman probe for the prospective in vivo clinical melanoma skin cancer screening. Rev. Sci. Instrum..

[24] Shen J., Zhao J., Jiang T., Li X., Zhao C., Su C., Zhou C. (2017). Predictive and prognostic value of folate receptor-positive circulating tumor cells in small cell lung cancer patients treated with first-line chemotherapy. Oncotarget.

[25] Krebs M.G., Sloane R., Priest L., Lancashire L., Hou J.-M., Greystoke A., Ward T.H., Ferraldeschi R., Hughes A., Clack G. (2011). Evaluation and prognostic significance of circulating tumor cells in patients with non-small-cell lung cancer. J. Clin. Oncol..

[26] Hirose T., Murata Y., Oki Y., Sugiyama T., Kusumoto S., Ishida H., Shirai T., Nakashima M., Yamaoka T., Okuda K. (2012). Relationship of circulating tumor cells to the effectiveness of cytotoxic chemotherapy in patients with metastatic non-small-cell lung cancer. Oncol. Res..

[27] Farace F., Massard C., Vimond N., Drusch F., Jacques N., Billiot F., Laplanche A., Chauchereau A., Lacroix L., Planchard D. (2011). A direct comparison of CellSearch and ISET for circulating tumour-cell detection in patients with metastatic carcinomas. Br. J. Cancer.

[28] Castle J., Morris K., Pritchard S., Kirwan C.C. (2017). Challenges in enumeration of CTCs in breast cancer using techniques independent of cytokeratin expression. PLoS ONE.

[29] Jie X.-X., Zhang X.-Y., Xu C.-J. (2017). Epithelial-to-mesenchymal transition, circulating tumor cells and cancer metastasis: Mechanisms and clinical applications. Oncotarget.

[30] Bitting R.L., Boominathan R., Rao C., Kemeny G., Foulk B., Garcia-Blanco M.A., Connelly M., Armstrong A.J. (2013). Development of a method to isolate circulating tumor cells using mesenchymal-based capture. Methods.

[31] Krebs M.G., Metcalf R.L., Carter L., Brady G., Blackhall F.H., Dive C. (2014). Molecular analysis of circulating tumour cells—Biology and biomarkers. Nat. Rev. Clin. Oncol..

[32] Hosokawa M., Kenmotsu H., Koh Y., Yoshino T., Yoshikawa T., Naito T., Takahashi T., Murakami H., Nakamura Y., Tsuya A. (2013). Size-Based Isolation of Circulating Tumor Cells in Lung Cancer Patients Using a Microcavity Array System. PLoS ONE.

[33] Dent B.M., Ogle L.F., Donnell R.L.O., Hayes N., Malik U., Curtin N.J., Boddy A.V., Plummer E.R., Edmondson R.J., Reeves H.L. (2016). Circulating Tumour Cells from Patients with Oesophageal, Hepatocellular, Thyroid and Ovarian. Cancers.

[34] Najjar F., Alammar M., Bachour M., Almalla N., Altahan M., Alali A., Al-Massarani G. (2015). Predictive and prognostic value of circulating endothelial cells in non-small cell lung cancer patients treated with standard chemotherapy. J. Cancer Res. Clin. Oncol..

[35] Weakley S.M., Wang H., Yao Q., Chen C. (2011). Expression and function of a large non-coding RNA gene XIST in human cancer. World J. Surg..

[36] Ma Z., Xue S., Zeng B., Qiu D. (2018). lncRNA SNHG5 is associated with poor prognosis of bladder cancer and promotes bladder cancer cell proliferation through targeting p27. Oncol. Lett..

[37] Fang J., Sun C.-C., Gong C. (2016). Long noncoding RNA XIST acts as an oncogene in non-small cell lung cancer by epigenetically repressing KLF2 expression. Biochem. Biophys. Res. Commun..

[38] Yu H., Xue Y., Wang P., Liu X., Ma J., Zheng J., Li Z., Li Z., Cai H., Liu Y. (2017). Knockdown of long non-coding RNA XIST increases blood–tumor barrier permeability and inhibits glioma angiogenesis by targeting miR-137. Oncogenesis.

[39] Wang H., Shen Q., Zhang X., Yang C., Cui S., Sun Y., Wang L., Fan X., Xu S. (2017). The long non-coding RNA XIST controls non-small cell lung cancer proliferation and invasion by modulating miR-186-5p. Cell. Physiol. Biochem..

[40] Raman C.V., Krishnan K.S. (1928). A New Type of Secondary Radiation. Nature.

[41] Deegan R.D., Bakajin O., Dupont T.F., Huber G., Nagel S.R., Witten T.A. (1997). Capillary flow as the cause of ring stains from dried liquid drops. Nature.

[42] Filik J., Stone N. (2007). Drop coating deposition Raman spectroscopy of protein mixtures. Analyst.

[43] Filik J., Stone N. (2008). Analysis of human tear fluid by Raman spectroscopy. Anal. Chim. Acta.

[44] Hale J.E. (2013). Advantageous Uses of Mass Spectrometry for the Quantification of Proteins. Int. J. Proteom..

[45] Simon K. (2016). Colorectal cancer development and advances in screening. Clin. Interv. Aging.

[46] Fiedor J., Fiedor L., Haessner R., Scheer H. (2005). Cyclic endoperoxides of beta-carotene, potential pro-oxidants, as products of chemical quenching of singlet oxygen. Biochim. Biophys. Acta.

[47] Nishino H., Tokuda H., Murakoshi M., Satomi Y., Masuda M., Onozuka M., Yamaguchi S., Takayasu J., Tsuruta J., Okuda M. (2000). Cancer prevention by natural carotenoids. Biofactors.

[48] Winnard P.T.J., Zhang C., Vesuna F., Kang J.W., Garry J., Dasari R.R., Barman I., Raman V. (2017). Organ-specific isogenic metastatic breast cancer cell lines exhibit distinct Raman spectral signatures and metabolomes. Oncotarget.

[49] Kong K., Kendall C., Stone N., Notingher I. (2015). Raman spectroscopy for medical diagnostics—From in-vitro biofluid assays to in-vivo cancer detection. Adv. Drug Deliv. Rev..

[50] Lewis P.D., Lewis K.E., Ghosal R., Bayliss S., Lloyd A.J., Wills J., Godfrey R., Kloer P., Mur L.A.J. (2010). Evaluation of FTIR Spectroscopy as a diagnostic tool for lung cancer using sputum. BMC Cancer.

[51] Holliday S.F., Kane-Gill S., Empey P.E., Buckley M., Smithburger P. (2014). Interpatient Variability in Dexmedetomidine Response: A Survey of the Literature. Sci. World J..

[52] Munzone E., Botteri E., Sandri M.T., Esposito A., Adamoli L., Zorzino L., Sciandivasci A., Cassatella M.C., Rotmensz N., Aurilio G. (2012). Prognostic value of circulating tumor cells according to immunohistochemically defined molecular subtypes in advanced breast cancer. Clin. Breast Cancer.

[53] Polyak K., Weinberg R.A. (2009). Transitions between epithelial and mesenchymal states: Acquisition of malignant and stem cell traits. Nat. Rev. Cancer.

[54] Beck J., Hennecke S., Bornemann-Kolatzki K., Urnovitz H.B., Neumann S., Ströbel P., Kaup F.-J., Brenig B., Schütz E. (2013). Genome Aberrations in Canine Mammary Carcinomas and Their Detection in Cell-Free Plasma DNA. PLoS ONE.

[55] Weiss G.J., Beck J., Braun D.P., Bornemann-Kolatzki K., Barilla H., Cubello R., Quan W.J., Sangal A., Khemka V., Waypa J. (2017). Tumor Cell-Free DNA Copy Number Instability Predicts Therapeutic Response to Immunotherapy. Clin. Cancer Res..

[56] Zeng X., Hood B.L., Zhao T., Conrads T.P., Sun M., Gopalakrishnan V., Grover H., Day R.S., Weissfeld J.L., Wilson D.O. (2011). Lung cancer serum biomarker discovery using label-free liquid chromatography-tandem mass spectrometry. J. Thorac. Oncol..

[57] Lewis A.T., Gaifulina R., Isabelle M., Dorney J., Woods M.L., Lloyd G.R., Lau K., Rodriguez-Justo M., Kendall C., Stone N. (2017). Mirrored stainless steel substrate provides improved signal for Raman spectroscopy of tissue and cells. J. Raman Spectrosc..

[58] Butler H.J., Ashton L., Bird B., Cinque G., Curtis K., Dorney J., Esmonde-White K., Fullwood N.J., Gardner B., Martin-Hirsch P.L. (2016). Using Raman spectroscopy to characterize biological materials. Nat. Protoc..

[59] Stone N., Kendall C., Smith J., Crow P., Barr H. (2004). Raman spectroscopy for identification of epithelial cancers. Faraday Discuss..

[60] Hutchings J., Kendall C., Shepherd N., Barr H., Stone N. (2010). Evaluation of linear discriminant analysis for automated Raman histological mapping of esophageal high-grade dysplasia. J. Biomed. Opt..

[61] Schirmer M.A., Beck J., Leu M., Oellerich M., Rave-Fränk M., Walson P.D., Schütz E., Canis M. (2018). Cell-Free Plasma DNA for Disease Stratification and Prognosis in Head and Neck Cancer. Clin. Chem..

[62] Kim D., Pertea G., Trapnell C., Pimentel H., Kelley R., Salzberg S.L. (2013). TopHat2: Accurate alignment of transcriptomes in the presence of insertions, deletions and gene fusions. Genome Biol..

[63] Trapnell C., Williams B.A., Pertea G., Mortazavi A., Kwan G., van Baren M.J., Salzberg S.L., Wold B.J., Pachter L. (2010). Transcript assembly and quantification by RNA-Seq reveals unannotated transcripts and isoform switching during cell differentiation. Nat. Biotechnol..

[64] Li H., Handsaker B., Wysoker A., Fennell T., Ruan J., Homer N., Marth G., Abecasis G., Durbin R. (2009). The Sequence Alignment/Map format and SAMtools. Bioinformatics.

[65] Pathan M., Keerthikumar S., Ang C.-S., Gangoda L., Quek C.Y.J., Williamson N.A., Mouradov D., Sieber O.M., Simpson R.J., Salim A. (2015). FunRich: An open access standalone functional enrichment and interaction network analysis tool. Proteomics.

[66] Gallart-Palau X., Serra A., Sze S.K. (2016). Enrichment of extracellular vesicles from tissues of the central nervous system by PROSPR. Mol. Neurodegener..

