# Supplementary material for: Liquid Biopsies in Lung Cancer: Four Emerging Technologies and Potential Clinical Applications
Source: Cancers (Basel). 2019 Mar 7;11(3):331. doi: 10.3390/cancers11030331 (PMC6468998; doi:10.3390/cancers11030331)
Supplement: Supplementary file 1 [file cancers-11-00331-s001.pdf]

Supplementary Materials: Liquid Biopsies in Lung Cancer: Four Emerging Technologies and Potential Clinical Applications

Dimple Chudasama, Periklis Katopodis, Nick Stone, Jennifer Haskell, Hannah Sheridan, Benjamin Gardner, Howard Urnowitz, Ekkehard Schuetz, Julia Beck, Marcia Hall, James Barr, Cristina Sisu, Alexandra Rice, Andreas Polychronis, Vladimir Anikin and Emmanouil Karteris

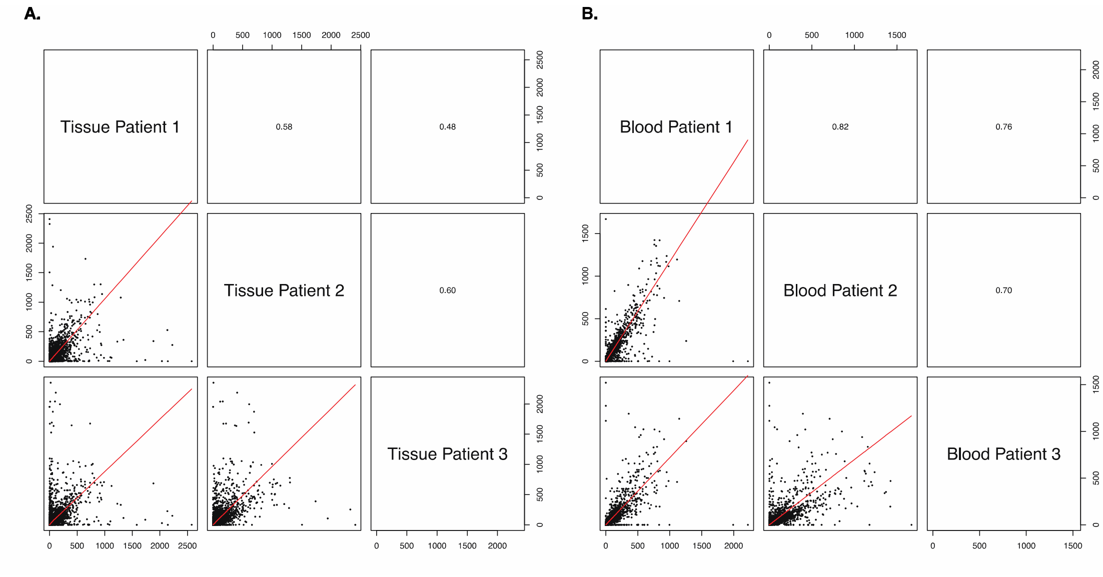

Figure S1. Cross patients' gene expression consistency. (**A**) Consistency in gene expression levels across tissue samples from three lung cancer patients. X and Y axis indicates absolute expression value in fragments per kilobase per million mapped reads (FPKM) for each gene. Pearson correlation coefficient for each pairwise comparison is given in the upper right-hand section of the matrix. (**B**) Consistency in gene expression levels across blood samples from three lung cancer patients. X and Y axis indicates absolute expression value in FPKM for each gene. Pearson correlation coefficient for each pairwise comparison is given in the upper right-hand section of the matrix.

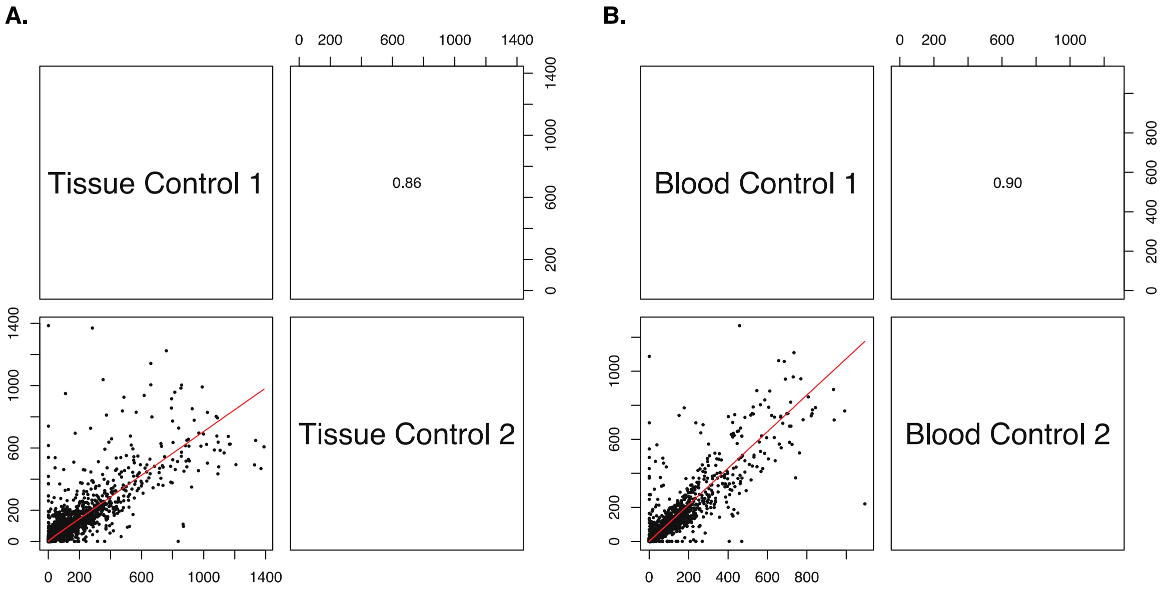

Figure S2. Cross control samples gene expression consistency. (**A**) Consistency in gene expression levels across lung tissue samples from two controls. X and Y axis indicates absolute expression value in FPKM for each gene. Pearson correlation coefficient for each pairwise comparison is given in the upper right-hand section of the matrix. (**B**) Consistency in gene expression levels across blood samples from two controls. X and Y axis indicates absolute expression value in FPKM for each gene. Pearson correlation coefficient for each pairwise comparison is given in the upper right-hand section of the matrix.

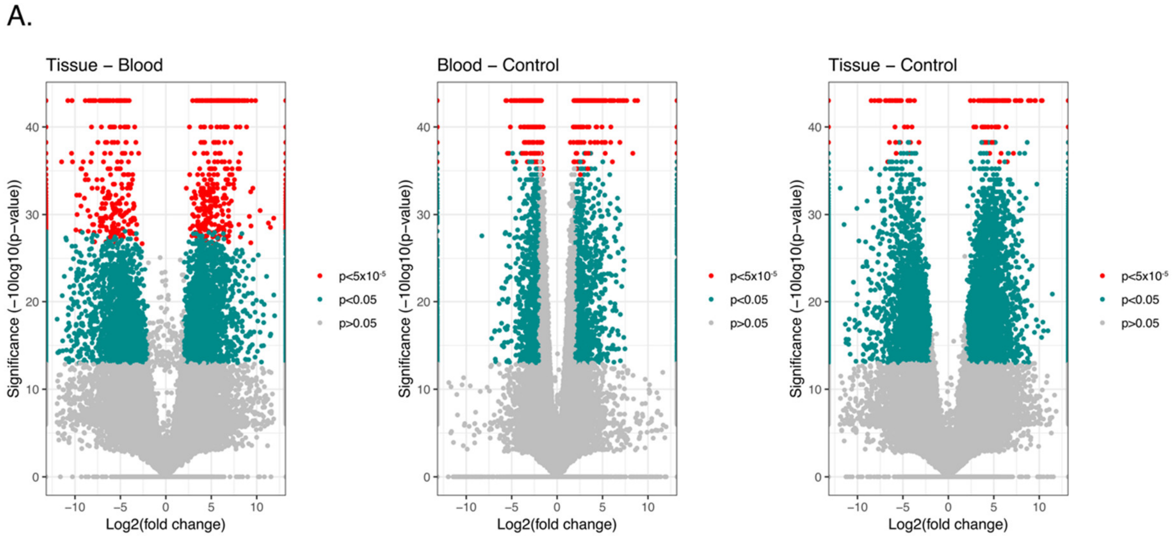

Supplementary Figure S3. *Cont.*

B.

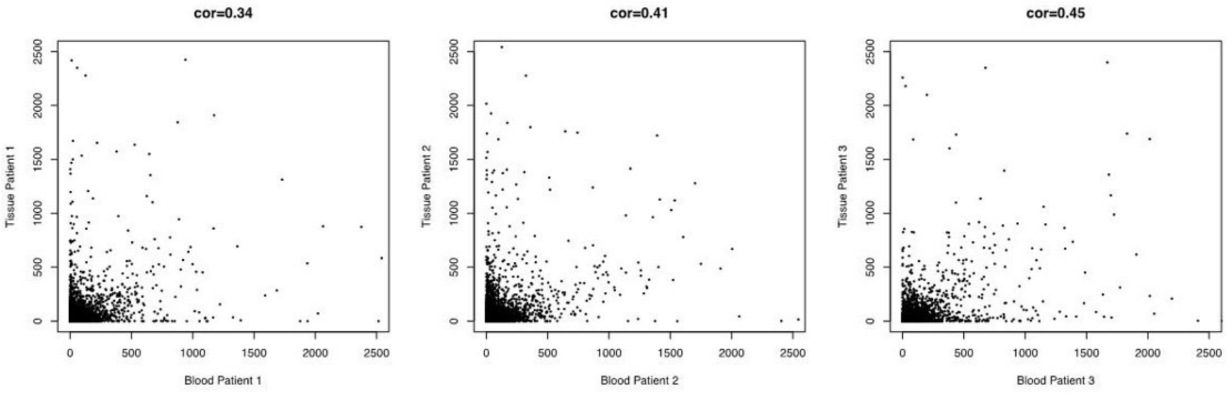

Figure S3. RNAseq gene expression analysis in tissue and blood. (**A**) Summary of differential expression patterns between LC patient tissue and blood (Tissue–Blood), LC patient blood and healthy blood controls (Blood–Control), and LC patient tissue and healthy tissue controls (Tissue–Control). Red dots indicate genes with a significant change in expression between the two analysed conditions ($p < 5 \times 10^{-5}$). Cyan dots indicate genes with a statistically significant ($p < 0.05$) change in expression between the two analysed conditions and a minimum fold change (FC) in expression (defined as of log2 (FC)), higher than 1. (**B**) Correlation coefficient between the gene expression levels in tissue and blood samples for each patient.

© 2019 by the authors. Licensee MDPI, Basel, Switzerland. This article is an open access article distributed under the terms and conditions of the Creative Commons Attribution (CC BY) license (http://creativecommons.org/licenses/by/4.0/).
